# Transcutaneous Afferent Patterned Stimulation for Essential Tremor: Real-World Evidence with Long Term Follow-Up

**DOI:** 10.5334/tohm.775

**Published:** 2023-08-29

**Authors:** Chiahao Lu, Dhira Khosla, Alexander Kent, Helen M. Bronte-Stewart, Kathryn H. Rosenbluth

**Affiliations:** 1 Cala Health Inc, San Mateo, CA, USA; 2 Stanford University School of Medicine, Stanford, CA, USA; 3 Department of Neurology and Neurological Sciences, Stanford University School of Medicine, Stanford, CA, USA

**Keywords:** peripheral neuromodulation, essential tremor, real-world evidence, remote monitoring, transcutaneous afferent patterned stimulation

## Abstract

**Background:**

Transcutaneous afferent patterned stimulation (TAPS) is a non-invasive neuromodulation therapy for the treatment of hand tremor in patients with essential tremor (ET). This retrospective post-market analysis evaluated the usage, effectiveness, and safety of TAPS in patients using TAPS beyond a 90-day trial period in a real-world setting.

**Methods:**

Study personnel screened a manufacturer’s database for TAPS devices that had been prescribed for the treatment of ET and used beyond a 90-day trial period between August 2019 and January 2023. The device logs were collected to extract the therapy usage, accelerometry measurements, and on-board ratings of tremor improvement. Study personnel also evaluated results of a voluntary survey requested by the manufacturer after the 90-day trial period. Adverse events were assessed from patients’ complaints reported to the manufacturer.

**Results:**

A total of 1,223 patients in the manufacturer’s database met the study criteria. The patients had used therapy between 90 and 1,233 days, with average usage of 5.6 sessions per week. Accelerometry data indicated 89% of patients experienced tremor improvement, with an average 64% improvement. 63% of patients rated at least half of their sessions as improved. No significant habituation was observed in patients who used therapy for more than one year. Approximately 62% of survey respondents either had reduced medication or planned to consult physicians about their medication usage. No serious safety events were reported, and 10% of patients reported minor safety complaints.

**Discussion:**

The analysis demonstrates the real-world effectiveness and safety of TAPS beyond a 90-day trial period over a longer timeframe and in a larger population size than previously published evidence.

## Introduction

Essential tremor (ET) is primarily characterized by persistent postural or kinetic tremor of the hands and forearms [1]. As the most common movement disorder [2], ET affects approximately 7 million Americans [3] and has been diagnosed in approximately 25 million patients worldwide [4]. ET often manifests with advancing age [5]. The common pathophysiology is associated with increased tremor-related activity in cerebellothalamocortical pathways [6,7,8]. ET affects a variety of activities of daily living, including eating, drinking, writing, and grooming, and many patients with ET report emotional burden, such as feeling embarrassed or stressed because of tremor [9]. This can result in a higher prevalence of depression and anxiety [10], leading to a significant decline in the patients’ overall health-related quality of life.

Treatments for ET include medications (e.g., propranolol and primidone), injections of botulinum toxin injections in the upper limb or neck [11], neurosurgical, and more recently, non-invasive neuromodulation approaches. Approximately half of patients with ET find that their tremor is medication-resistant or that the side effects from medication are intolerable [12]. Thalamic deep brain stimulation or thalamotomy via radiofrequency or focused ultrasound has shown benefit in patients with advanced ET [11], however these surgical interventions carry substantial cost [13] and surgical risks [14].

A non-invasive neuromodulation intervention was developed to address the proportion of patients with ET who find themselves in the treatment gap between medications and surgical options. Transcutaneous afferent patterned stimulation (TAPS) is delivered by an FDA-cleared, wrist-worn neurostimulation device that measures each patient’s unique physiology and delivers an alternating stimulation signal to the median and radial peripheral nerves. The device is calibrated to each patient’s tremor frequency [15,16,17,18,19]. Prescriptions for TAPS therapy can accompany pharmacotherapy or be used without pharmacological therapies [20]. TAPS is self-administered as needed by patients, typically when patients’ tremors are worse or in advance of an activity for which tremor control is desired, such as eating. Patients receive an initial TAPS therapy kit which includes training materials, a stimulator, a charging base station, and a wrist band with embedded electrodes to be used for the first 90-days of training and trial. After the initial 90 days, new wrist bands are required for patients who continue to use TAPS therapy. Training and trial periods are common for other neuromodulation therapies including spinal cord stimulation and sacral nerve stimulation [21,22], and generally provide a window of time for patients to develop real-world experience with when and how to use therapy, and allow physicians to assess each patient’s therapeutic response and provide guidance on each patient’s therapy usage.

A previous real-world analysis of TAPS safety and effectiveness in patients using TAPS beyond the 90-day trial period reported results from 321 patients, most of whom had used TAPS for less than a year [19]. This analysis aims to update and extend the real-world evidence on TAPS with a broader cohort of patients that have used TAPS beyond the 90-day trial period, including individuals with over three years of TAPS usage.

## Methods

### Patient Selection

**Figure 1:**
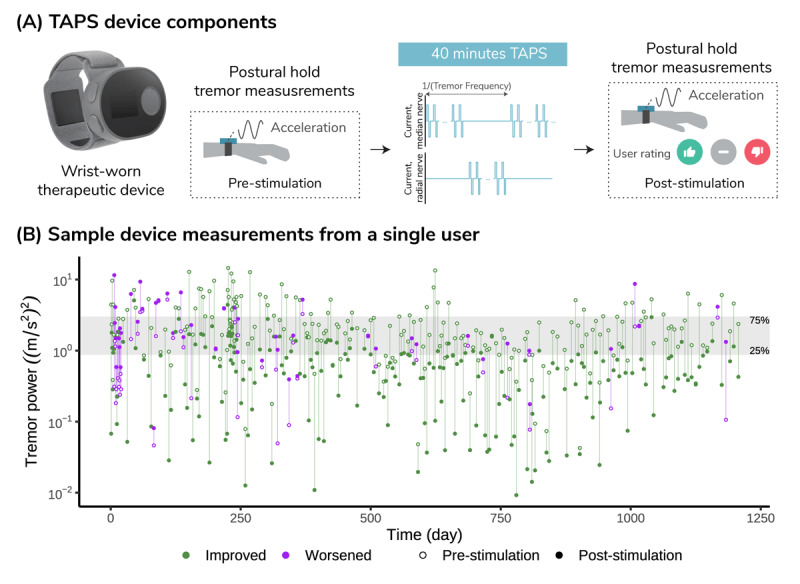
**TAPS device and example data.** (A) The wrist-worn TAPS device is equipped with a stimulator and band containing stimulation electrodes. For each therapy session, stimulation is delivered for 40-min as alternating bursts of stimulation pulses alternating between median and radial nerves at the patient’s calibrated tremor frequency. Patients are prompted to perform postural holds for tremor measurement and ratings (*improved, no change*, or *worsened*) before and after each of the 40 sessions and every 7^th^ session thereafter. (B) Example tremor power measurements from a long-term TAPS patient. Each point denotes a measurement from a single postural hold with open circles representing pre-stimulation and filled circles for post-stimulation. The green color denotes improved tremor after a single session while purple indicates worsened tremor. The display is logarithmic and the lowest (25^th^ percentile) and highest (75^th^ percentile) quartiles of pre-stimulation tremor power are shown as lower and upper bounds of light gray shaded area. This patient observed more improvement after approximately 500 days of usage, suggesting the patient might have learned when to apply TAPS therapy to reach the most beneficial effect.

This retrospective analysis screened a TAPS device manufacturer’s database for patients that had been prescribed TAPS to treat ET and had used TAPS beyond a 90-day trial period between August 1^st^, 2019, to January 31^st^, 2023 (device: Cala Trio™; manufacturer: Cala Health, San Mateo, CA, USA, Figure 1A), following the methods established in a previous real-world analysis [19]. The inclusion criteria for all analyses were as follows: 1) the prescribing healthcare provider indicated a diagnosis of ET (ICD-10 code G25.0) on the patient’s TAPS prescription form; and 2) the patient had used TAPS beyond the 90-day trial period, as identified by the time lapse between the patients first and last TAPS sessions. The exclusion criterion for all analyses, except the survey, was that patients had not completed an insufficient number of sessions, defined as having completed fewer than 10 sessions that were at least 20 minutes in duration. While a complete TAPS session was 40 minutes in duration, a minimum therapy duration of 20 minutes was selected because patients could discontinue a session at any time and a prior study suggested patients experienced therapeutic benefit after the first 20 minutes of stimulation [18]. Additionally, patients were excluded from the effectiveness analysis if they did not have at least 10 sessions of TAPS that a) included accelerometry measurements that were free of motion artifact and could be used to measure effectiveness before and after therapy, b) included accelerometry measures that were measured no longer than 15 minutes before or 15 minutes after therapy, and c) were started at least 120 minutes after a prior session (to minimize carry-over effects). For patients prescribed two devices for bilateral tremor, only the device with more sessions was included in the analysis.

### Data Collection

Demographic data, device data, survey results and device complaints were collected as described previously [19]. Briefly, demographic and tremor history information was collected from the patient’s prescription records. Timestamps of device usage, accelerometry data, and self-rated change in tremor severity were collected from device logs in the manufacturer’s centralized secured database. Three options (i.e., *improved, no change*, or *worsened*) for change in tremor severity were prompted on board the device because a more complex rating (e.g., full clinical global impression scale) would be more burdensome to the user. Figure 1B provides an example of session data derived from the device log of a single patient. Survey responses were collected from the voluntary surveys sent to all device users 90 days after they started therapy (see Table S1 for survey questions). Analysis of adverse events was performed using device complaints reported to the manufacturer.

### Usage Analysis

Therapy usage was assessed as the 1) range of usage periods across patients, measured in days; 2) percentage of patients who used therapy for at least 360 days and 720 days; and 3) average number of sessions per week for different usage periods (≤180 days, >180 days, >360 days and >720 days) within two cohorts (age < 65 years and age ≥ 65 years). The usage period was defined as the time interval between the first and last session for each patient.

### Effectiveness Analysis

Therapy effectiveness was evaluated using 1) tremor power improvement ratio, 2) percentage of tremor reduction, and 3) percentage of sessions rated as improved. Tremor power was calculated as the integral of the accelerometry data power spectral density in a window around the peak tremor frequency between 4–12 Hz (see [17,18] for more detailed descriptions of tremor power). Per-session tremor power improvement ratio was calculated as the ratio of tremor power of the pre-stimulation postural hold divided by the tremor power of the post-stimulation postural hold. A tremor power improvement ratio over 1 signifies improved tremor, whereas a value under 1 represents worsened tremor. Percentage of tremor reduction was calculated for each therapy session as follows:


\[
Tremor\;Reduction\left( \% \right) = \frac{{Tremor\;powe{r_{pre - stimulation}} - Tremor\;powe{r_{post - stimulation}}}}{{Tremor\;powe{r_{pre - stimulation}}}} \times 100
\]

Percentage of sessions with tremor power improvement ratio greater than 1 and median tremor power improvement ratio across all sessions were calculated for each patient. Additionally, tremor power improvement ratio and percentage of sessions rated as improved were calculated using two different classifications. Specifically, the calculations were based on tremor power from pre-stimulation postural holds using 1) within-subject classification, to evaluate improvement within individual subjects, and 2) between-subject classification, to evaluate improvement across the population. For the within-subject category, each session performed by an individual patient was classified into high (>75^th^ percentile), medium (25^th^ to 75^th^ percentile) and low (<25^th^ percentile), based on the pre-stimulation tremor power across all sessions performed by that patient. Subsequently, three values (for high, medium, and low classifications) were calculated per patient for the percentage of sessions with tremor power improvement ratio greater than 1 and for median tremor power improvement ratio. For the between-subject category, each patient was classified into high, medium, and low based on the median pre-stimulation tremor power taken from all sessions in each individual patient. Thus, for the between-subject category, only one value was calculated per patient for percentage of sessions with tremor power improvement ratio greater than 1 and for median tremor power improvement ratio.

Potential habituation of TAPS therapy was investigated in patients who used therapy for at least 360 days. Habituation was assessed using the median tremor improvement ratio of each 90-day usage period through 360 days, as well as any usage beyond 360 days for each patient.

The effectiveness of repeated (back-to-back) sessions was assessed to identify whether extended sessions maintained tremor improvement. A repeated session was defined as stimulation sessions separated by less than 10 minutes from the end of the first to the start of the second session. To evaluate the effectiveness of repeated sessions, median tremor power improvement ratios for 1) pre-stimulation of the first session to post-stimulation of the second session (pre1/post2); 2) pre-stimulation of the first session to post-stimulation of the first session (pre1/post1) and 3) pre-stimulation of the second session to post-stimulation of the second session (pre2/post2) were evaluated for repeated sessions.

### Safety Complaints Analysis

Device safety was assessed by analyzing complaints reported to the manufacturer. The type and frequency of adverse events reported as complaints were extracted from the manufacturer’s complaint database.

### Statistical Analysis

Device usage, accelerometry data, device prompted self-rated tremor severity change, complaints, and survey data were all summarized and reported in descriptive statistics.

To investigate how pre-stimulation tremor severity affected improvement, a linear mixed effects regression model was used for each classification respectively, with tremor power improvement ratio as the dependent variable, patient as the random-intercept term, and pre-stimulation tremor severity as the fixed factor. To determine how tremor improvement varied over time to assess potential habituation of TAPS effects, a linear mixed effects regression model was used with the same dependent variable and random-intercept term, and time period (levels: days 0–90, 91–180, 181–270, 271–360) as the fixed factor. The tremor improvement ratio was transformed to logarithmic scale before entering the mixed models to account for skewness [23] and the average value was summarized descriptively across patients as geometric mean unless otherwise stated. Significance was tested with *F*-tests (Satterthwaite’s degrees of freedom method) with Tukey adjustment for pairwise comparisons in mixed models.

For repeated sessions analysis, one sample *t*-tests (one-tailed) were performed to evaluate if each of the three log-transformed tremor improvement ratios were greater than 0, i.e., original tremor power improvement ratio greater than 1. Holm-Bonferroni adjustment was used to correct for performing multiple *t*-tests. Two-tailed *p*-value threshold was set at 0.05 with one-tailed *p*-value set to 0.025. All statistical analyses were performed in R (version 4.2.1).

## Results

### Data Availability

A total of 1,223 patients in the manufacturer’s database met the inclusion and exclusion criteria for this retrospective analysis. The patients had an average age of 73 years and had been prescribed TAPS by healthcare providers trained in neurology (47.9%), family practice (17.3%) and internal medicine (14.0%) (Table 1). Among the 1,223 patients meeting the criteria for the usage analysis, 808 passed the exclusion criteria for the effectiveness analysis. Eighty-one patients were excluded from the effectiveness analysis for having no postural hold data. Another 334 patients were excluded for having fewer than ten sessions of postural hold data, following the sequential order of criteria requiring a) accelerometry measurements free of motion artifact (N = 127), b) accelerometry measurements no more than 15 minutes before or after therapy (N = 47), and c) sessions started at least 120 minutes after a prior session (N = 160).

**Table 1:** Population characteristics.


DEMOGRAPHICS	

Total number	1,223

Age ≥ 65 years	1,037

Sex, men^1^	72.7%

Age (years)	73.0 ± 9.7

**TAPS PRESCRIBER SPECIALTY^2^**	

Neurology	47.9%

Family medicine	17.3%

Internal medicine	14.0%

Occupational or physical therapist	2.9%

Surgery (Neurosurgery included)	0.7%

Psychiatry	0.5%

Cardiology	0.1%

Other^3^	16.0%

**PATIENT-REPORTED TREMOR BURDEN^4^**	

Years with tremor symptoms	

<5 years	15.1%

5–10 years	36.5%

10–20 years	22.2%

>20 years	26.2%

Self-rated tremor severity prior to TAPS	

Mild	3.2%

Moderate	63.5%

Marked	28.6%

Severe	4.8%

Number medications tried prior to TAPS	

None	15.9%

1	25.4%

2	32.5%

3	9.5%

4 or more	16.7%

Number of current medications for tremor	

None	35.7%

1	41.3%

2	23.0%

3	0%

4 or more	0%

Most important area of therapeutic need	

Activities of daily living	72.2%

**PATIENT-REPORTED TREMOR BURDEN^4^**	

Social activities	4.8%

Hobbies	6.3%

Professional responsibilities	15.9%

Housework	0.8%

**PATIENT-REPORTED USAGE CHARACTERISTICS**	

Activity level during TAPS therapy	

Normal	39.7%

Perform some activities with hand	24.6%

Hand movement limited	24.6%

No hand movement while sitting still	11.1%

Usage case	

Acute	52.4%

Preventative	47.6%


Categorical data reported as percentage; Continuous data reported as mean ± 1 standard deviation. ^1^ From 1,208 patients with sex data available. ^2^ From 851 TAPS prescribers of 1,223 patients. ^3^ Examples of others include emergency medicine and anesthesiology. ^4^ From 126 survey respondents.

### Usage Analysis

The usage analysis included 260,207 TAPS sessions performed by the 1,223 patients. The duration of usage ranged from 90 days (the minimum requirement to meet the study’s inclusion criteria) to 1,223 days. Patients performed an average of 5.6 TAPS sessions per week (Table 2). Patients over the age of 65 years (84.8% of patients) had approximately one more weekly therapy session compared to those under 65 (≥65 years, 5.7 vs <65 years, 4.8 sessions per week). Weekly usage was similar across time periods (≤180, >180, >360, and >720 days; range 5.4 to 5.9 sessions per week).

**Table 2:** Descriptive statistics of weekly TAPS usage.


	ALL PATIENTS	AGE < 65 YEARS	AGE ≥ 65 YEARS

Usage period (day)^1^			

≤180	5.4 ± 4.0 (383)	4.8 ± 3.6 (55)	5.5 ± 4.1 (328)

>180	5.6 ± 4.0 (840)	4.8 ± 3.7 (131)	5.8 ± 4.0 (709)

>360	5.6 ± 3.9 (422)	4.9 ± 2.8 (60)	5.7 ± 4.1 (362)

>720	5.9 ± 3.7 (92)	4.5 ± 2.6 (10)	6.1 ± 3.8 (82)

All	5.6 ± 4.0 (1,223)	4.8 ± 3.7 (186)	5.7 ± 4.1 (1,037)


Data reported as mean ± 1 standard deviation with number of patients in parentheses. ^1^ Usage period defined as time interval (day) between the first valid therapy session (stimulation duration ≥20 mins) and the last valid session for each patient.

Of patients who had total usage beyond 360 days (i.e., those that passed the 90-day trial period for the study at least 270 days prior to the data pull), 60.9% (422 of 693 qualified patients) had continued to use therapy. Of patients who had total usage beyond 720 days (i.e., those that passed the 90-day trial period for the study at least 630 days prior to the data pull), 54.8% (92 of 168 qualified patients) continued to use therapy.

### Effectiveness analysis

Effectiveness data from 36,411 sessions performed by the 808 patients passing the exclusion criteria were available for analysis. The average (geometric mean) tremor power improvement ratio across all patients was 2.8 (arithmetic mean, 710.5; median, 2.1), i.e., 64.3% reduction in tremor power. In addition, 49.8% of patients showed at least 50% of tremor reduction, and 88.1% of patients had at least 50% of sessions with tremor improvement ratio greater than 1 (Figures 3A and 3C). The supplementary material includes additional analysis of the percentages of sessions with tremor improvement ratio greater than the minimal detectable change.

**Figure 2:**
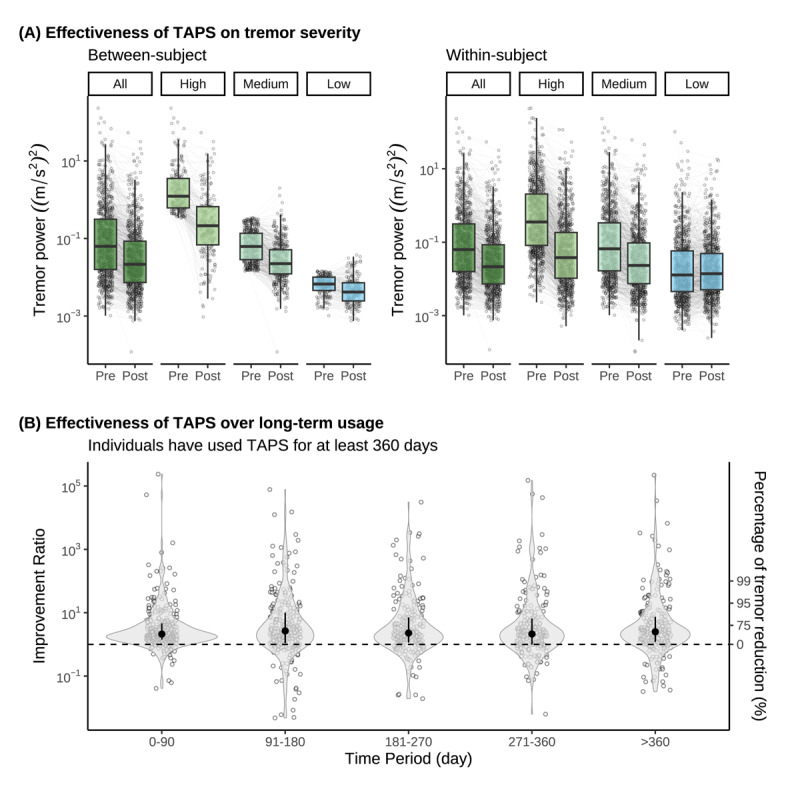
**TAPS effectiveness outcomes and severity categorization.** (A) Tremor power at pre- and post-stimulation across All, High, Medium, and Low groups for both between-subject (population) and within-subject classifications. Each point denotes the tremor power from a single session, with a boxplot superimposed to show group median (thick black line), interquartile range (upper and lower bound of each box) and 1.5 × interquartile range (upper and lower whiskers). Sessions and patients in the high group improved the most in comparison to other groups, while the tremor power remained relatively mild in for the low group; (B) Tremor power improvement ratio over different time periods in long-term patients who used TAPS for at least 360 days. Each open circle denotes the tremor power improvement ratio computed from postural holds from a single session with a violin plot showing the distribution and black filled circle with vertical bars representing median with interquartile ranges. No statistically significance was found in the median improvement ratios across different time periods, suggesting no habituation of TAPS therapy for tremor improvement.

**Figure 3:**
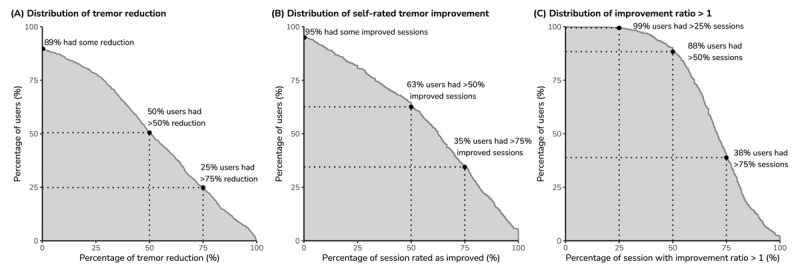
**Objectively measured and subjectively perceived tremor improvement.** (A) Across all sessions, the cumulative distribution of per-patient median tremor power reductions indicated the vast majority (89%) of patients improved and most (50%) experienced meaningful improvement greater than 50% (N = 808 patients). (B) Across all sessions with available patient ratings, the cumulative distribution of per-patient percentage of sessions with self-rated tremor improvement indicated most (63%) of patients meaningfully improvement on most sessions (N = 805 patients); (C) Across all sessions, the cumulative distribution per-patient percentage of sessions with tremor power improvement ratio greater than 1 indicated the vast majority (88%) of patients had some tremor improvement in the majority of their sessions.

A significant main effect of pre-stimulation tremor severity was observed in both classifications (within-subject: *F*_(2, 35606)_ = 4734.7, *p* < 0.001; between-subject: *F*_(2, 816.28)_ = 106.05, *p* < 0.001) (Table 3). Figure 2A demonstrates the change in tremor power before and after each therapy session for both classifications. Specifically, patients with the high quartile tremor severity in both within-subject (average improvement ratio: 9.6; 95% CI, 8.8 to 10.4) and between-subject (average improvement ratio: 7.5; 95% CI, 5.8 to 7.8) classification experienced the most tremor improvement compared with patients with the low or medium tremor severity (*p* < 0.001 for all post-hoc pairwise comparisons in both classifications).

**Table 3:** Descriptive statistics of TAPS effectiveness outcomes.


	WITHIN-SUBJECT	BETWEEN-SUBJECT (POPULATION)

Tremor power improvement ratio^1^		

High tremor severity	9.6 (9.0)	7.5 (17.0)

Medium tremor severity	2.8 (7.6)	2.3 (7.0)

Low tremor severity	0.9 (7.4)	1.5 (5.3)

All	2.8 (9.3)	

Sessions rated as improved^2^		

High tremor severity	59.6%	65.5%

Medium tremor severity	61.1%	62.8%

Low tremor severity	60.4%	51.2%

All	60.5%	

Sessions rated as no change^2^		

High tremor severity	37.7%	32.3%

Medium tremor severity	36.4%	34.6%

Low tremor severity	37.7%	46.0%

All	37.1%	

Sessions rated as worsened^2^		

High tremor severity	2.7%	2.4%

Medium tremor severity	2.5%	2.2%

Low tremor severity	1.9%	2.8%

All	2.4%	


^1^ Data reported as geometric mean with 1 geometric standard deviation in parentheses. Geometric standard deviation represents ×/÷ factor change from geometric mean. ^2^ Data from 33,847 sessions in 805 patients where the self-rated post-stimulation tremor severity change was available. Note, the tremor severity level was determined using pre-stimulation tremor power, more details are provided in the methods section for each level.

No habituation was observed in TAPS effectiveness in long-term patients, based on analysis of 17,527 sessions in 303 patients with device usage of at least 360 days (Figure 2B). The average tremor power improvement ratio was similar across all time periods, ranging from 3.1 to 3.7 (67.7% to 73.0% reduction in tremor power). No significant main effect of time period (*F*_(4, 17406)_ = 2.14, *p* = 0.072) was found (see Figure 2B).

Three patients did not complete the session ratings, leaving 33,847 sessions by 805 of the patients available for analysis of ratings. On average, 60.5% of sessions were rated as improved. The percent of sessions rated as improved were comparable across the within-subject classification (about 60%) while the average percentages were greater in high and medium groups compared with the low group in the between-subject classification (Table 3). The average percentage of sessions rated as no change was 37.1%, and 2.4% of sessions were rated as worsened (Table 3). The results on self-ratings showed 63.4% of patients had at least 50% of sessions rated as improved (Figure 3B).

A total of 628 paired therapy sessions from 223 patients were included in the analysis of repeated sessions. Due to the limited number of repeated sessions and in keeping with previous analysis [19], additional repeated sessions (e.g., the 3^rd^ session of 3 consecutive sessions) were excluded from the analysis. The average improvement ratio was 2.9 (65.5% reduction in tremor power) from pre-stimulation of the first session to post-stimulation of the second session. This was constituted by an average improvement ratio of 2.6 (61.5% reduction in tremor power) from pre- to post-stimulation of the first session (pre1/post1), and 1.4 (28.6% reduction in tremor power) from pre- to post-stimulation of the second session (pre2/post2). All log-transformed tremor improvement ratios were significantly greater than 0 (pre1/post2: *t*_(222)_ = 7.44, *p* < 0.001, lower bound of 97.5% CI, 0.79; pre1/post1: *t*_(222)_ = 7.94, *p* < 0.001, lower bound of 97.5% CI, 0.73; pre2/post2: *t*_(222)_ = 2.41, *p* = 0.008, lower bound of 97.5% CI, 0.06).

### Survey analysis

**Figure 4:**
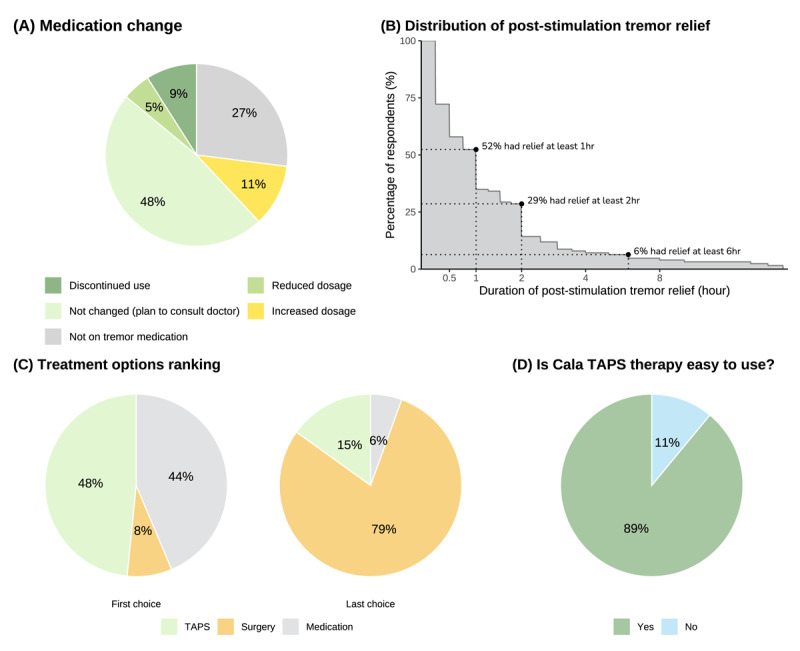
**Patient-reported survey outcome.** (A) Approximately 62% of survey respondents either had reduced medication (11%), or planned to consult physicians to reduce medication (48%); (B) Cumulative distribution of per-patient self-perceived tremor improvement duration after TAPS therapy, suggesting many respondents obtain prolonged tremor improvement after stimulation; (C) Respondents’ order rankings of preference for different therapy options, showing patients generally preferred TAPS over medication or surgical management of tremor; (D) Most respondents indicated TAPS therapy is easy to use.

In this analysis, 1,998 patients received an email with a link to the survey and voluntary survey responses from 126 patients (6.3% response rate) were available for analysis (Table 1). Approximately 84% of patients had tried at least 1 tremor medication and 27% had tried at least 3 tremor medications prior to TAPS usage. After using TAPS, 14% of patients either reduced medication dosage or completely discontinued medication, while 48% of respondents planned to consult their physicians to consider changing medication usage (Figure 4A). Figure 4B shows that over half of the patients reported their tremor improvement persisted at least for an hour after TAPS therapy and 29% of patients indicated benefit of at least 2 hours following therapy, with some patients experiencing tremor improvement beyond 6 hours. Just over half of respondents indicated a preference for TAPS over medication or surgical intervention for tremor management, while just under half of patients indicated a preference for medication and few patients indicated a preference for surgery (Figure 4C). Most respondents (89%) found the device easy to use (Figure 4D). About half of the respondents applied TAPS in a preventative manner and about half applied TAPS for acute relief (Table 1). Most patients (72%) identified activities of daily living as the most important need for therapy (Table 1).

### Safety Analysis

At least one safety-related complaint was reported in 10.2% of patients, including discomfort (e.g., electric shock, burning, pain, tingling, numbness, or swelling; 5.3% of patients), skin irritation (itchiness, redness, or rash; 6.9% of patients) at or near the stimulation site, and physical symptoms (e.g., discomfort, pain, or stiffness outside of the stimulation site, headache, altered vision; 1.9% of patients). No serious safety events were reported.

## Discussion

This multi-year retrospective analysis reports findings on usage, effectiveness, and safety of TAPS therapy in patients using therapy beyond a 90-day trial period from a real-world setting. A previous real-world data analysis has suggested TAPS is an effective and safe therapy to alleviate tremor in patients with ET [19]. This analysis shows consistent findings in a greater sample size (1,223 patients for therapy usage analysis and 808 patients for effectiveness analysis) and extends the usage period up to 3.4 years, confirming and extending evidence for the durability of TAPS effectiveness over long-term usage.

Most patients in the analysis were over age 65, consistent with the reported prevalence of ET [4]. Prescriptions for the use of TAPS were most frequently written by physicians trained as neurologists, with additional prescribers including a variety of medical professionals such as internal medicine physicians, family medicine physicians, and rehabilitation therapists.

This real-world dataset demonstrates that patients who used TAPS beyond a 90-day trial period used TAPS therapy slightly less than once per day. This usage was maintained reliably over time, with patients using TAPS therapy 5.4 sessions per week during the first 180 days versus 5.6–5.9 sessions per week beyond 180 days. The results also suggest that 61% of the patients who use therapy beyond a 90-day trial period continue using TAPS beyond one year and that more than half of patients continue using TAPS beyond two years. This suggests adherence to TAPS may be stronger than adherence to ET medications [24], despite the additional effort associated with using and charging a device over the ease of taking an oral medication.

The effectiveness of TAPS demonstrated in this analysis was similar to that observed in a previous clinical study and real-world evidence analysis [17,19]. In this analysis of 36,411 sessions from 808 patients, TAPS reduced tremor severity by 64% (average improvement ratio: 2.8) and half of patients had at minimum two-fold improvement in tremor power (Figure 3A). This expands upon previously published real-world findings, in which tremor power was reduced by 71% across 6,048 treatment sessions in 216 patients, and over half of patients experienced a two-fold or better reduction in tremor following their treatment sessions [19]. Broadly, these results are also in alignment with sham-controlled acute findings [16] and those of a 3-month prospective trial clinical trial in which tremor power was reduced by more than half for 54% of subjects [17]. Another report showed that tremor reduction could persist for one hour following treatment sessions [18], which is consistent with the subjective report of long-lasting therapeutic benefits in this analysis (Figure 3B) and may afford patients extended time in which to perform activities of daily living.

Patients gain the most benefit when TAPS is used in a more severe tremor state (e.g., Figure 2A, within-subject, high severity). This holds true when analyzed both across the patient population with various tremor severities, and within a single patient’s sessions. Although mild tremor intrinsically cannot be improved as much as severe tremor due to a potential floor effect, TAPS can still maintain or slightly improve mild tremor (e.g., Figure 2A, within-subject, low severity). In line with a prior real-world analysis [19], no habituation was observed in this analysis of 303 patients who used TAPS for at least one year. In fact, patients may achieve more tremor improvement over time by learning when to use TAPS for greatest benefit, as demonstrated in the long-term patient shown in Figure 1B. Furthermore, this analysis indicates that delivering back-to-back therapy sessions may extend tremor improvement. The median tremor power improvement ratio, relative to the start of the first session, was 2.6 after the first session and 2.9 after the second session, suggesting tremor improvement was persistent over repeated sessions. Future studies are warranted to prospectively examine whether delivery of repeated TAPS therapy sessions could prolong the duration of tremor improvement.

Complications associated with device usage, which included skin irritation and minor discomfort, were generally mild and resolved with topical treatments or cessation of device use. The number of patients who reported adverse events, and the types of adverse reported, were consistent with prior clinical studies and the previous real-world data analysis [17,19].

Remote data collection enabled by the TAPS device provides the opportunity to assess patients’ real-world tremor severity, therapy usage and effectiveness, and potentially, progression of the underlying ET disease state. These data may benefit prescribing physicians as well as patients to inform clinical decision-making and optimize treatment benefits.

There were several limitations to this analysis that should be acknowledged. First, the analysis only evaluated patients using TAPS for longer than a 90-day trial period. While this is consistent with other neurostimulation devices that are evaluated during a training and trial period ahead of continued use, such as spinal cord stimulation and sacral nerve stimulation [21,22], it limits generalization of current findings for users who discontinued use during the 90-day trial. Reasons for discontinuation during the 90-day trial are attributable to many factors that are beyond the scope of this manuscript, including patients without access to insurance coverage who may return therapy before the end of their 90-day trial period. Second, data collection relied on patients’ device utilization and adherence to performing postural holds, as well as the voluntary completion of survey data. Therefore, the current findings may not be reflective of all patients’ experience. Future research could be strengthened by incorporating video recordings of postural holds that are scored by blinded raters. Third, although a previous study using the same wrist-worn accelerometer demonstrates significant correlation between clinical tremor rating and tremor power from accelerometry for postural tremor [17], using the wrist-worn accelerometry could only provide a proxy assessment of hand tremor amplitude. Together with the potential floor effect, this might explain why patients with relatively mild tremor severity would not improve as much due to the insensitivity of the sensor location. Fourth, only adverse events that were reported to the manufacturer by the patients were recorded, which may have led to under-reporting of the adverse event rate. Fifth, because some survey questions were designed for patients who continue to use TAPS beyond a 90-day trial period, these may have been posed with an inherent bias towards indicating improvement. For example, the question supporting Figure 4B may have been asked in a way that indicated improvement after stimulation. Finally, anonymous survey response collection made it impossible to associate responses with individual respondents or determine if they met the inclusion criteria for this analysis. Furthermore, the low response rate to the voluntary survey could have biased the results. Despite these limitations, the analysis validates real-world use patterns and outcomes that are complementary to clinical trials, which can have limited generalization to clinical practice [25,26].

In conclusion, this analysis reinforces and extends prior findings on safety, usage, and durable effectiveness of TAPS for tremor management in patients with ET using multi-year data. A sizable portion of patients who use TAPS beyond a 90-day trial period continue to use TAPS to alleviate tremor even past two years of usage, implying that these patients still receive treatment benefits from the device, in line with the sustained effectiveness observed in this long-term real-world monitoring. Finally, patients reported a slight preference for TAPS over medication and a strong preference over surgery.

## Data Accessibility Statements

The data that support the findings of this study are available from the corresponding author, [CL], upon reasonable request.

## Additional File

The additional file for this article can be found as follows:

10.5334/tohm.775.s1 Supplementary material. Table S1. Survey questions & minimum detectable change analysis.
