# Supplementary material for: Transcutaneous Afferent Patterned Stimulation for Essential Tremor: Real-World Evidence with Long Term Follow-Up
Source: Tremor Other Hyperkinet Mov (N Y). 2023 Aug 29;13:29. doi: 10.5334/tohm.775 (PMC10473165; doi:10.5334/tohm.775)
Supplement: Supplementary material. — Table S1. Survey questions & minimum detectable change analysis. [file tohm-13-1-775-s1.pdf]

Supplementary material

Table S1. Survey questions

For how many years have you had hand tremor symptoms?

- Less than 5 years
- 5-10 years
- 10-20 years
- Greater than 20 years

How would you rate the overall tremor severity of your treated hand without using Cala Trio?

- Mild – mild tremor not causing difficulty in performing any activities
- Moderate – tremor causes difficulty in performing some activities
- Marked – tremor causes difficulty in performing most or all activities
- Severe – tremor prevents performing some activities

How many tremor medications have you tried prior to starting Cala Trio?

- None
- 1
- 2
- 3
- 4 or more

How many tremor medications are you currently taking for your hand tremor?

- None
- 1
- 2
- 3
- 4 or more

Have you changed your tremor medication dosage since starting Cala Trio?

- Discontinued use
- Reduced dosage
- Increased dosage
- Not changed, but plan to consult my doctor
- I am not on tremor medications

If Cala Trio were presented as an option at the same time as medications or surgical procedures, which would you choose? (rank in order;1 = would choose first; 3 = would choose last)

- Cala Trio
- Surgical procedures
- Tremor Medications

Is Cala Trio easy to use?

- Yes
- No

How do you generally use Cala Trio?

- On a consistent schedule, regardless of how severe my tremor is at that time
- At varying times whenever my tremor seems worse or bothers me more

What do you do during your 40-minute Cala Trio therapy session?

- I go about my normal activities.
- I do some activities with my hand.
- I try to limit my hand movement.
- I sit still and do not move my hand.

Which activities would you most like Cala Trio therapy to help you with? (rank in order of importance; 1 = most important to you, 4 = least important to you)

- Activities of daily living (e.g., eating, drinking)
- Social activities
- Hobbies
- Professional responsibilities/work
- Housework

On average, how long did tremor relief last after a stimulation session?

- 15minutes
- 30 minutes
- 45 minutes
- 1 hour
- 1 hour 15 minutes
- 1 hour 30 minutes
- 1 hour 45 minutes
- 2 hours
- 2 hour 30 minutes
- 3 hours
- 3 hour 30 minutes
- 4 hours
- 5 hours
- 6 hours
- 8 hours
- 10 hours
- 18 hours
- 21 hours
- 24 hours+

Analysis of percentages of improved sessions using minimal detectable change

To account for potential measurement noise in reporting the percentage of sessions with tremor power improvement ratio greater than 1, an additional analysis was performed to estimate the minimal detectable change (MDC) based on the pre-stimulation tremor power. Previous studies have shown that MDC could be calculated from the within-subjects residual mean squared error (within-subjects variability) in a repeated-measures analysis of variance (ANOVA) (1,2). However, due to the nature of this retrospective real-world analysis, a repeated-measures ANOVA would appear inappropriate due to the unbalanced design, i.e., different number of measurements across subjects In contrast, mixed models can account for an unbalanced design and use all available observations (3).

A mixed effects model was built with log-transformed post-stimulation tremor power as the dependent variable, log_{10}-transformed pre-stimulation tremor power as the fixed effect, and individual patient as the random effect. Then, the 95% confidence interval (CI) of the log_{10}-transformed pre-stimulation tremor power was estimated from this model with a parametric bootstrap method of 1,000 iterations. The absolute difference between the 95% CI was calculated as the MDC. Finally, improvement in each session was defined as having a change between pre-and post-stimulation tremor power (both log_{10}-transformed) greater than the MDC.

The effect of log-transformed pre-stimulation tremor power was statistically significant and positive (beta = 0.31; 95% CI, [0.297, 0.318]; $t_{(34475)} = 58.1$; $p < 0.001$). The model's intercept (log_{10}-transformed pre-stimulation tremor power equal to 0) was at -1.18 (95% CI, [-1.22, -1.14]; $t_{(36407)} = -58.07$; $p < 0.001$). The MDC value was 0.021, equivalent to the original tremor power improvement ratio at 1.05 (back transformed from log_{10}-transformation value of 0.021). The results show that 86.5% of patients experienced at least 50% of sessions with improvement greater than the MDC value and the median percentage of improved sessions is 68.8% (IQR, 21.7%; mean, 68.4%; SD, 16.3%) across the cohort.

References

1. Weir JP. Quantifying Test-Retest Reliability Using the Intraclass Correlation Coefficient and the SEM. Journal of Strength and Conditioning Research. 2005;19(1):231–40.

2. Elble RJ, McNames J. Using Portable Transducers to Measure Tremor Severity. Tremor and Other Hyperkinetic Movements. 2016 May 17;6(0):375.

3. Detry MA, Ma Y. Analyzing Repeated Measurements Using Mixed Models. JAMA. 2016 Jan 26;315(4):407.
